# Elimination of hydrogenase active site assembly blocks H_2_ production and increases ethanol yield in *Clostridium thermocellum*

**DOI:** 10.1186/s13068-015-0204-4

**Published:** 2015-02-12

**Authors:** Ranjita Biswas, Tianyong Zheng, Daniel G Olson, Lee R Lynd, Adam M Guss

**Affiliations:** Biosciences Division, Oak Ridge National Laboratory, Oak Ridge, TN 37830 USA; BioEnergy Science Center, Oak Ridge National Laboratory, Oak Ridge, TN 37830 USA; Thayer School of Engineering at Dartmouth College, Hanover, NH 03755 USA; Current address: Centre for Rural Development and Technology, Indian Institute of Technology Delhi, Hauz Khas, New Delhi, 110016 India; One Bethel Valley Road, Oak Ridge, TN 37831-6038 USA

**Keywords:** Cellulosic ethanol, *Clostridium thermocellum*, Hydrogenase maturation, Metabolic engineering

## Abstract

**Background:**

The native ability of *Clostridium thermocellum* to rapidly consume cellulose and produce ethanol makes it a leading candidate for a consolidated bioprocessing (CBP) biofuel production strategy. *C. thermocellum* also synthesizes lactate, formate, acetate, H_2_, and amino acids that compete with ethanol production for carbon and electrons. Elimination of H_2_ production could redirect carbon flux towards ethanol production by making more electrons available for acetyl coenzyme A reduction to ethanol.

**Results:**

H_2_ production in *C. thermocellum* is encoded by four hydrogenases. Rather than delete each individually, we targeted hydrogenase maturase gene *hydG*, involved in converting the three [FeFe] hydrogenase apoenzymes into holoenzymes. Further deletion of the [NiFe] hydrogenase (*ech*) resulted in a mutant that functionally lacks all four hydrogenases. H_2_ production in *∆hydG∆ech* was undetectable, and the ethanol yield nearly doubled to 64% of the maximum theoretical yield. Genomic analysis of *∆hydG* revealed a mutation in *adhE*, resulting in a strain with both NADH- and NADPH-dependent alcohol dehydrogenase activities. While this same *adhE* mutation was found in ethanol-tolerant *C. thermocellum* strain E50C, *∆hydG* and *∆hydG∆ech* are not more ethanol tolerant than the wild type, illustrating the complicated interactions between redox balancing and ethanol tolerance in *C. thermocellum*.

**Conclusions:**

The dramatic increase in ethanol production suggests that targeting protein post-translational modification is a promising new approach for simultaneous inactivation of multiple enzymes.

**Electronic supplementary material:**

The online version of this article (doi:10.1186/s13068-015-0204-4) contains supplementary material, which is available to authorized users.

## Background

A sustainable future will likely be dependent on large-scale production of fuels, chemicals, and products from renewable resources. One of the most promising approaches to this end is microbially catalyzed conversion of lignocellulosic biomass [1]. Abundant plant biomass resources are available that have the potential to be used as feedstocks [2], but economical bioconversion of plant material into fuel has been elusive [3]. Microbes may ultimately play a central role in the conversion of biomass to fuels and chemicals. While current technologies for biomass fermentation to fuels tend to rely on added cellulolytic enzymes to solubilize hemicellulose and cellulose prior to fermentation, enzyme production represents a significant cost and hinders economic production of biofuels [4]. An alternative approach is to utilize one or more microorganisms to ferment plant cell walls to fuels in one step without added enzymes; this process is called consolidated bioprocessing (CBP). However, no known naturally occurring microbe is capable of robust, high yield, and high titer fuel production from lignocellulose. Thus, genetic modification will be required to create an organism with all of the desired properties for bioconversion of lignocellulosic biomass to fuels.

*Clostridium thermocellum*, a thermophilic, cellulolytic member of the Firmicutes phylum, is a potential platform to engineer into a CBP organism due to its native ability to efficiently solubilize cellulose and produce ethanol as a fermentation product. However, wild-type *C. thermocellum* is limited by its low ethanol yield and titer, producing acetate, lactate, H_2_, formate, and free amino acids as additional fermentation products [5], and native strains tolerate low levels of ethanol [6,7]. Recently, methodologies for genetic manipulation have been developed for *C. thermocellum* [8-12], raising the possibility that it can be engineered to economically produce fuels from cellulosic substrates. While these tools are still laborious to use, they have now been successfully used to improve industrially important properties in *C. thermocellum*. For instance, ethanol tolerance has been correlated with mutations in the bifunctional acetaldehyde/alcohol dehydrogenase, *adhE*, in three independent strains of *C. thermocellum* [6,13], and heterologous expression of a mutant *adhE* gene in an otherwise wild-type strain conferred ethanol tolerance [13]. Metabolic flux to major end products has also been blocked via gene deletion, including production of acetic acid [11], lactic acid in an *adhE* mutant [14], and acetic and lactic acid simultaneously [8], increasing ethanol yield. *C. thermocellum* does not encode a pyruvate kinase and is instead thought to divert significant flux from phosphoenolpyruvate → oxaloacetate → malate → pyruvate in what is called a malate shunt, which would generate NADPH while oxidizing NADH to NAD^+^, possibly creating a redox imbalance [15-17]. Heterologous expression of pyruvate kinase and disruption of the malate shunt in *C. thermocellum* also substantially improved flux towards ethanol [16], but further increases in ethanol yield are still needed.

While acetic and lactic acids are the primary soluble fermentation products competing with ethanol synthesis, H_2_ production is a major sink for electrons that could otherwise be directed toward ethanol production. By using protons as an electron sink rather than glycolytic intermediates, acetyl coenzyme A (acetyl-CoA) becomes available for production of acetate and ATP. While this provides more usable energy for the cell, it decreases the pool of electrons available for reduction of acetyl-CoA.

**Figure 1:**
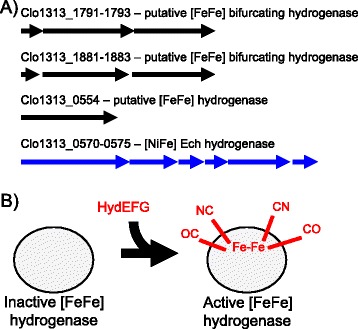
***C. thermocellum*** **hydrogenases and hydrogenase maturation. A)** Chromosomal loci of *C. thermocellum* encoding three [FeFe] hydrogenases (in black) and one [NiFe] hydrogenase (in blue). **B)** Together, HydE, HydF, and HydG assemble the active site of [FeFe] hydrogenases to make active enzymes.

H_2_ production is catalyzed by a class of enzymes called hydrogenases, which broadly fall into three primary categories based on the metals in the active site: [Fe] hydrogenases, which are thus far only found in methanogens, [FeFe] hydrogenases, and [NiFe] hydrogenases. *C. thermocellum* encodes three putative [FeFe] hydrogenases and one ferredoxin-dependent [NiFe] energy-converting hydrogenase (Ech) (Figure 1A, [18,19]). Hydrogenase active sites are complex organometallic catalysts that require a dedicated enzymatic system for post-translational assembly. [FeFe] hydrogenases utilize a single system for active site assembly, consisting of the maturases HydE, HydF, and HydG (Figure 1B, reviewed in [20]). HydF acts as a scaffold upon which the binuclear Fe active site is assembled. HydE likely produces the ligand that bridges the two active site Fe molecules, while HydG cleaves tyrosine to generate the -CN and -CO ligands on the active site Fe molecules. Each is required for [FeFe] hydrogenase activity, and thus represents a novel target for simultaneous inactivation of multiple hydrogenases. We hypothesized that targeting electron flux to H_2_ can be a fruitful approach to increasing flux to ethanol. Therefore, we targeted inactivation of *hydG* as part of a strategy to eliminate H_2_ as a fermentation product and redirect metabolic flux toward ethanol.

## Results

### Hydrogenase maturase deletion simplifies elimination of H_2_ as a fermentation product

**Figure 2:**
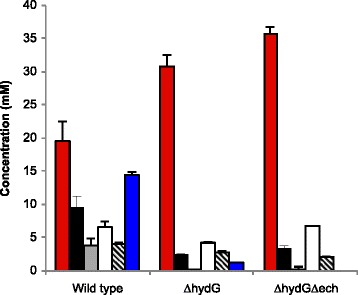
**Fermentation products of** ***C. thermocellum*** **.** The wild-type, *∆hydG*, and *∆hydG∆ech* strains grown on minimal medium with 5 g/L cellobiose. Red bar, ethanol; black bar, acetate; gray bar, lactate; white bar, formate; diagonal line bar, total amino acids; blue bar, H_2_. H_2_ concentration is reported as mmol gaseous H_2_ per L liquid medium to facilitate comparison to soluble products. Error bars represent one standard deviation.

To redirect electrons away from H_2_ and toward ethanol, we deleted the [FeFe] hydrogenase maturase gene *hydG* to prevent conversion of the hydrogenase apoenzymes into holoenzymes. Because HydEFG is only involved in maturation of the [FeFe] hydrogenases, we further deleted the genes encoding the [NiFe] Ech hydrogenase (Additional file 1). Deletion of *hydG* dramatically decreased H_2_ production, with a 15-fold reduction (Figure 2). Further deletion of *ech*, resulting in strain *ΔhydGΔech*, completely eliminated detectable H_2_ production and verified that deleting *hydG* simultaneously eliminated all [FeFe] hydrogenase activity.

**Figure 3:**
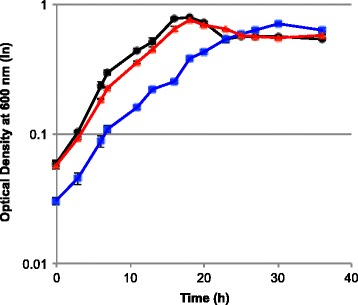
**Growth profile of** ***C. thermocellum*** **strains on minimal medium.** Symbols: Black circles, wild-type *C. thermocellum*; blue squares , *C. thermocellum ∆hydG*; red triangles , *C. thermocellum ∆hydG∆ech*. Error bars represent one standard deviation.

In anaerobic batch fermentation, the *ΔhydG* mutant, which was later found to also contain a point mutation in the bifunctional acetaldehyde/alcohol dehydrogenase *adhE* (Clo1313_1798) (see below), produced 63% more ethanol than the parent strain (Figure 2). It decreased acetate production by 74%, lactate production was nearly eliminated, formate production decreased by 34% compared to the wild type, and total secreted amino acid levels decreased as well. Further deletion of *ech* in the *∆hydG* background completely eliminated H_2_ production, while ethanol production increased by 90% compared to the wild type and 16% relative to *∆hydG*. This represents an overall ethanol yield from cellobiose of 64% of the theoretical yield. The maximum optical density was 12% lower in *∆hydG* (OD600 = 0.70) relative to the wild type (OD600 = 0.80), and the growth rate was also slower (wild type = 0.26 h^−1^, *∆hydG* = 0.12 h^−1^). The growth yield and rate of *ΔhydGΔech* were between these values, with a maximum OD600 = 0.76 and a growth rate of 0.22 h^−1^ (Figure 3).

**Figure 4:**
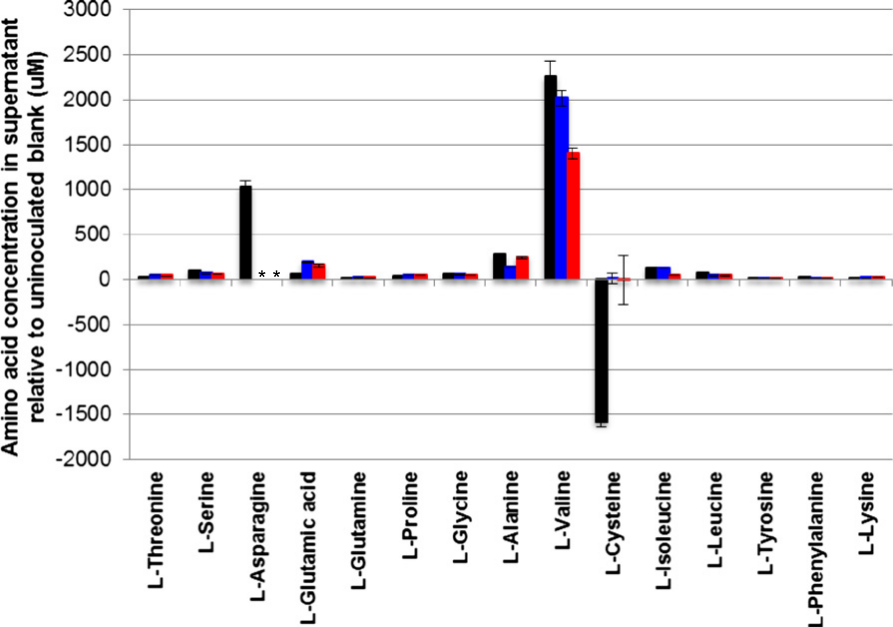
**Concentration of amino acids produced by wild-type,** ***ΔhydG***, **and** ***∆hydG∆ech*** **strains.** *, asparagine quantification for two samples was prevented by interference. Error bars represent one standard deviation. Black bars, wild-type *C. thermocellum*; blue bars, *C. thermocellum ∆hydG*; red bars, *C. thermocellum ∆hydG∆ech*.

As *C. thermocellum* is known to divert a significant flux toward production of secreted amino acids, we further examined the abundance of individual amino acids in the supernatant. Among the other secreted amino acids, L-valine was produced in significant amounts in all the strains (Figure 4) as previously observed [21], but was 38% lower in *ΔhydGΔech*. Interestingly, the wild-type strain removed about 1.6 mM of 2.4 mM cysteine in the medium, while *C. thermocellum ΔhydG* and *ΔhydGΔech* did not consume any cysteine from the medium (Figure 4).

### Mutation in *adhE* altered cofactor specificity but not ethanol tolerance

During strain validation, a point mutation in the bifunctional acetaldehyde/alcohol dehydrogenase *adhE* (Clo1313_1798) was discovered in *C. thermocellum ∆hydG*, converting aspartate 494 into glycine (D494G). This exact same mutation was identified in the ethanol-tolerant *C. thermocellum* mutant E50C [6], though the effect of this mutation has not been validated enzymatically or genetically. A different mutant AdhE from the ethanol-tolerant strain C. thermocellum adhE* (EA) containing two mutations (P704L and H734R) was found to alter coenzyme specificity, eliminating NADH-dependent acetaldehyde reduction but allowing NADPH-dependent acetaldehyde reduction [13]. We therefore tested the effect of the D494G mutation on ADH cofactor specificity in *C. thermocellum ∆hydG*. While the wild-type ADH specific activity was almost exclusively NADH-dependent (7.03 ± 1.06 μmol/min/mg protein with NADH; 0.10 ± 0.08 μmol/min/mg protein with NADPH), ADH specific activity was high with each cofactor in *C. thermocellum ∆hydG* (6.31 ± 1.27 μmol/min/mg protein with NADH; 5.89 ± 0.89 μmol/min/mg protein with NADPH). As a control, the specific activity of ADH from the C. thermocellum adhE* (EA) strain containing the P704L and H734R mutations was tested, resulting in 0.006 ± 0.007 μmol/min/mg protein NADH-dependent activity and 0.25 ± 0.005 μmol/min/mg protein NADPH-dependent activity, consistent with previous results [13]. Because previously identified *adhE* mutations in *C. thermocellum*, including D494G, correlated with an ethanol tolerance phenotype [6,13], we examined the effect of added ethanol on the growth of *C. thermocellum ∆hydG*. In rich (Additional file 2, left) and minimal media (Additional file 2, right), *∆hydG* and *∆hydG∆ech* did not display an ethanol-tolerant phenotype.

## Discussion

Genetic manipulation is possible in an increasing number of Firmicutes, and tremendous progress in these systems has been made in recent years. However, depending on the gene and bacterium, the effort to delete a single gene can be time-consuming and a barrier to advanced metabolic engineering approaches. Here, we identified a commonality between multiple desired enzymatic targets and designed a strategy to target them simultaneously. Targeting post-translational modification machinery might be a generally useful approach to streamline metabolic engineering under certain circumstances. Particularly in H_2_-producing organisms such as *Clostridium cellulolyticum* (three predicted [NiFe] hydrogenases; four predicted [FeFe] hydrogenases), *Clostridium acetobutylicum* (two predicted [FeFe] hydrogenases), and *Clostridium beijerinckii* (six predicted [FeFe] hydrogenases) [18], this same approach of targeting hydrogenase maturation to divert flux away from H_2_ + acetate or butyrate is quite promising. It may also enable fundamental studies of H_2_ metabolism in organisms such as sulfate-reducing bacteria [22-24].

**Figure 5:**
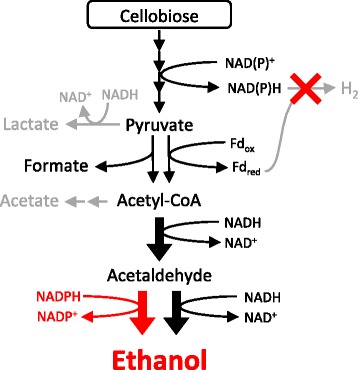
**Overview of metabolic changes in** ***C. thermocellum ΔhydGΔech*** **.** When hydrogenases were inactivated (red X), flux to ethanol increased. Further, the alcohol dehydrogenase mutated, allowing use of NADPH as a cofactor for ethanol production (red pathway). Simultaneously, fluxes to H_2_, lactate, and acetate decreased or were eliminated (gray pathways).

Altering electron flux via elimination of H_2_ production had a dramatic effect on carbon flux in *C. thermocellum* (Figure 5). Previous *C. thermocellum* metabolic engineering efforts primarily focused on altering carbon flux directly; deletion of phosphotransacetylase (*pta*) nearly eliminated acetate as a fermentation product but had little impact on ethanol yield [11]. Similarly, deletion of *pta* and lactate dehydrogenase together only increased ethanol yield slightly to 27% of the theoretical yield, but strain evolution allowed for increased conversion up to 59% of the theoretical yield [8]. Heterologous expression of a pyruvate kinase and blockage of the malate shunt via deletion of malic enzyme resulted in an ethanol yield of 47% of the theoretical yield [16]. Instead of targeting carbon flux directly, we constrained electron flux by both blocking [FeFe] hydrogenase activity and deleting the Ech hydrogenase. Combined with the spontaneous mutation in *adhE*, this dramatically reduced production of acetate and eliminated H_2_, resulting in the highest yet achieved ethanol yield in *C. thermocellum,* 64% of the theoretical maximum without yet evolving the strain for improved performance. The impact of the *adhE* mutation on product yields in these strains is currently unclear, but the evolved strain E50C [6,7] contains the exact same point mutation, and the ethanol yield is comparable to that of the wild type. While *ΔhydG* grew slowly, likely due to difficulty balancing redox reactions, it attained a similar maximum OD to that of the wild type (Figure 3). The metabolic load of producing hydrogenase apoproteins could contribute to the diminished growth rate, especially if they are overproduced in response to the *ΔhydG* mutation, but the hydrogenases were expressed in *ΔhydG* and *ΔhydGΔech* at the same level as in the wild type (CM Wilson, unpublished data), suggesting that metabolic load does not play a significant role in the growth defect. Further deletion of *ech* allowed for a growth rate more similar to that of the wild type while also redirecting more flux to ethanol. This demonstrates that *C. thermocellum* is capable of sufficiently rerouting metabolism to accommodate the lack of H_2_ production.

Production of acetic acid plus CO_2_ is obligately coupled to production of a more reduced compound, either H_2_ or formate in *C. thermocellum*, in order to balance redox reactions. Thus, decreasing or eliminating H_2_ production without altering acetate production would result in a redox imbalance, and decreasing flux through this pathway could help alleviate this imbalance. Therefore, the decrease in acetate production seen here is expected upon the elimination of H_2_ production. While elimination of the [FeFe] hydrogenases alone increased ethanol yield substantially, the additional removal of the Ech hydrogenase was needed to further increase yield, clearly demonstrating the importance of Ech in this system. This result might be unexpected, because previous proteomics studies showed a lack of Ech protein in the proteome of related strain *C. thermocellum* ATCC27405 [25]. The cause of this discrepancy might well be the difficulty of detecting membrane proteins during proteomics studies, differences between *C. thermocellum* strains ATCC27405 and DSM1313, or differences in physiology due to the deletion of *hydG* in our study.

The hydrogenase mutants also stopped synthesizing lactate via lactate dehydrogenase (*ldh*). In *C. thermocellum* Ldh is known to be allosterically activated by fructose-1,6-bisphosphate (FbP) [26], presenting an alternate mechanism by which lactate production could have been eliminated. Perhaps the hydrogenase deletion strains have low levels of FbP, preventing Ldh from being active. Further, FbP-activated Ldh enzymes from other anaerobic thermophiles are also regulated by other metabolites, such as inhibition by NADPH in *Thermoanaerobacter ethanolicus* [27] and inhibition by pyrophosphate and activation by ATP in *Caldicellulosiruptor saccharolyticus* [28]. While these potential allosteric regulators have not been tested on the *C. thermocellum* Ldh, deletion of the hydrogenases would likely result in an accumulation of reduced ferredoxin via pyruvate: ferredoxin oxidoreductase (PFOR). This reduced ferredoxin plus NADH could then be used as substrates by NfnAB (Clo1313_1848 - 1849) to transfer electrons to NADPH [29], potentially resulting in low levels of NADH and an overabundance of NADPH, leading to inhibition of Ldh. With decreased Ldh activity and an AdhE capable of using both NADH and NADPH, the flux to lactate will decrease and flux to ethanol would then naturally increase to regenerate the cofactors.

Previous characterization of an ethanol-tolerant mutant of *C. thermocellum* revealed that it contained, among many other mutations, two point mutations in *adhE*, resulting in amino acid changes P704L and H734R. This mutation was reconstructed in an otherwise wild-type strain, resulting in altered alcohol dehydrogenase (ADH) enzyme cofactor specificity from NADH-dependent to NADPH-dependent and increased ethanol tolerance [13]. This suggested that an unknown redox imbalance occurs as the ethanol concentration increases. Additional evolved ethanol-tolerant *C. thermocellum* strains E50C and E50A also acquired independent mutations in *adhE*, resulting in amino acid changes to D494G and G553R, respectively [6]. However, the effect of the latter two mutations in an otherwise wild-type strain has not yet been determined. Here, the *hydG* mutant independently acquired the exact same D494G mutation as strain E50C. Unlike the previous P704L-H734R mutant, NADH-dependent activity was not eliminated. Instead, the *hydG* deletion strain contained high levels of both NADH- and NADPH-dependent ADH activities. Interestingly, *ΔhydG* was not ethanol tolerant, illustrating the complexity of the *C. thermocellum* ethanol tolerance phenotype. While it is possible that the D494G mutation does not contribute to the ethanol tolerance phenotype of strain E50C, a more likely explanation is that the additional redox imbalance(s) caused by deletion of *hydG* prevent the D494G mutation from rebalancing metabolism. Regardless, this gain of function mutation that allows use of either NADH or NADPH for reduction of acetaldehyde to ethanol will be useful for further metabolic engineering efforts by minimizing NADH/NADPH redox imbalances. Indeed, *C. thermocellum ΔhydGΔech*, with its high ethanol yield, near wild-type growth rate, and ADH capable of utilizing both NADH and NADPH, is now an ideal platform for further engineering of *C. thermocellum* for production of cellulosic fuels and chemicals.

## Conclusions

While *Clostridium thermocellum* degrades cellulose well, efforts to engineer this strain are laborious and time-consuming. In addition to making ethanol, *C. thermocellum* converts cellulosic to lactate, formate, acetate, H_2_, ethanol, amino acids, and other products that must be eliminated to achieve high yield. Here, we developed a methodology to simultaneously inactivate multiple enzymes by targeting a common post-translational modification system, simplifying strain construction by minimizing the necessary genetic modifications. We eliminated H_2_ production and successfully redirected electron and carbon flux towards desired product. This strain will serve as a platform for further engineering to economically produce fuels and chemicals from lignocellulosic biomass.

## Materials and methods

### Strains and culture conditions

Standard molecular methods were used for plasmid cloning [30,31]. *Escherichia coli* TOP10 and BL21 were grown in LB medium supplemented with 12 μg ml^−1^ chloramphenicol when appropriate. *Clostridium thermocellum* DSM1313 and mutant strains were grown in a modified DSM122 medium similar to that described by Tripathi *et al.* [11] supplemented with 5 μg ml^−1^ thiamphenicol (TM; Sigma-Aldrich, St. Louis, MO, USA), 50 μg ml^−1^ 5-fluoro-2′-deoxyuridine (FUdR, Sigma-Aldrich, St. Louis, MO, USA), and 500 μg ml^−1^ 8-azahypoxanthine (8AZH; Acros Organics, Pittsburgh, PA, USA) as needed and MTC minimal medium [32] prepared as described in [6]. The modified DSM122 medium composition was (L^−1^): 3 g sodium citrate tribasic dehydrate, 1.3 g ammonium sulfate, 1.43 g potassium phosphate monobasic, 1.8 g potassium phosphate dibasic trihydrate, 0.5 g cysteine-HCl, 10.5 g 3-morpholino-propane-1-sulfonic acid (MOPS), 6 g glycerol-2-phosphate disodium, 0.41 g sodium acetate, 5 g cellobiose, 4.5 g yeast extract, 0.13 g calcium chloride dehydrate, 2.6 g magnesium chloride hexahydrate, 0.0011 g ferrous sulfate heptahydrate, and 0.0001 g resazurin, adjusted to pH 7.0. The minimal medium consisted of (L^−1^): 2 g sodium citrate dehydrate, 1.25 g citric acid monohydrate, 1 g sodium sulfate, 1 g potassium phosphate dibasic trihydrate, 2.5 g sodium bicarbonate, 1.5 g ammonium chloride, 2 g urea, 1 g magnesium chloride hexahydrate, 0.2 g calcium chloride dehydrate, 0.1 g ferrous chloride tetrahydrate, 1 g L-cysteine hydrochloride monohydrate, 5 g cellobiose, 0.001 g resazurin, 5 g MOPS, 20 mg pyridoxamine dihydrochloride, 1 mg riboflavin, 1 mg nicotinamide, 0.5 mg DL-thioctic acid, 4 mg 4-amino benzoic acid, 4 mg D-biotin, 0.025 mg folic acid, 2 mg cyanocobalamin, 4 mg thiamine hydrochloride, 0.5 mg MnCl_2_.4H_2_O, 0.5 mg CoCl_2_.6H_2_O, 0.2 mg ZnSO_4_.7H_2_O, 0.05 mg CuSO_4_.5H_2_O, 0.05 mg HBO_3_, 0.05 mg Na_2_MoO_4_.2H_2_O, and 0.05 mg NiCl_2_.6H_2_O.

### Plasmid and strain constructions

Strains were constructed as previously described [8-10] in strain *C. thermocellum ∆hpt*, which is a derivative of strain DSM1313 and is herein referred to as wild type. Plasmid pAMG278 (Additional file 3) was used to delete *hydG* (Clo1313_1571)*,* resulting in strain *C. thermocellum ∆hpt ∆hydG*, hereafter referred to as *C. thermocellum ∆hydG*. In the *C. thermocellum ∆hydG* background, plasmid pAMG275 (Additional file 3) was used to delete the [NiFe] maturase and hydrogenase enzymes *hypABFCDE* (Clo1313_0564 to 0569) and *echABCDEF* (Clo1313_0570 to 0575)*,* resulting in strain *C. thermocellum ∆hpt ∆hydG ∆hyp-ech*, hereafter referred to as *C. thermocellum ∆hydG∆ech*. All genetic manipulation was carried out using the modified DSM122 medium. Briefly, the plasmids were each isolated from a *dcm*^*−*^ strain of *E. coli* (Guss et al., 2012) [9], transformed into *C. thermocellum ∆hpt* via electroporation, and plated in medium supplemented with TM. Colonies were picked into liquid medium with TM, grown at 51°C, and plated in medium supplemented with TM and FUdR. Colonies were single colony purified and picked into liquid medium with TM. These cultures were then subcultured without TM and plated with 8AZH to select for the final deletion, followed by single colony purification to obtain pure cultures [8-10]. Deletion strains were confirmed by PCR (Additional file 1; primer sequences in Additional file 4). Strain purity was further confirmed by 16S rRNA gene sequencing.

### Fermentation conditions

The inoculum for batch fermentation was prepared by growing the mutants in MTC medium overnight at 55°C in an anaerobic chamber (COY Laboratory Products, Inc., Grass Lake, MI, USA). The fermentation was carbon limited and carried out in 27-ml Balch tubes with 10 ml of MTC media containing 5 g L^−1^ of cellobiose under a N_2_ headspace sealed with butyl rubber stoppers. The tubes were inoculated with 0.5% v/v culture and incubated at 55°C. The fermentation products were determined after 53 hours of growth. The final cellobiose concentration was usually less than 0.5 mM, suggesting that fermentation activity was complete. Fermentations were performed at least two times with three independent biological replicates each. Growth rate was calculated using the change in absorbance during mid-log phase, from an OD600 of approximately 0.1 to 0.3.

### Ethanol tolerance

Ethanol tolerance was tested in Balch tubes containing minimal medium with 0, 1, 2, 3, 4, and 5% v/v added ethanol, inoculated with 0.5% of overnight grown culture, and incubated at 55°C. Growth was monitored by measuring the optical density at 600 nm on a Unico 1200 spectrophotometer (Unico, Dayton, NJ, USA). Growth experiments were performed at least two times with three independent biological replicates each.

### Analytical methods

Fermentation products, including ethanol, acetate, lactate, and formate, were analyzed on a Breeze 2 High Performance Liquid Chromatograph system using an Aminex-HPX-87H column with a 5 mM sulfuric acid mobile phase. H_2_ was measured using an Agilent Technologies 6850 Series II Gas Chromatograph (Agilent, Santa Clara, CA, USA) using a thermal conductivity detector at 190°C with a N_2_ reference flow and a Carboxen 1010 PLOT (30.0 m × 530 μm I.D.; model Supelco 25467) column.

### Enzyme assay of alcohol dehydrogenase (ADH)

50-ml cultures of *C. thermocellum* cells were grown anaerobically to OD_600_ = 0.3 and harvested by centrifugation at 2800 × g for 30 minutes at 4°C and stored at −80°C. Before the assay, the pellet was resuspended anaerobically in 0.5 ml Assay Buffer (0.1 M Tris–HCl, 0.1 mM Fe_2_^+^, 0.1 mM DTT, pH 7.5). The cells were lysed with 2 μl Ready-Lyse Lysozyme from Epicentre Technologies (Madison, WI, USA), and 1 μl DNase I from New England Biolabs (Ipswich, MA, USA) was added to reduce viscosity. The resulting solution was centrifuged at 17,000 × g for 10 minutes at room temperature, and the supernatant was used as cell-free extracts for enzyme assays. For the ADH (acetaldehyde reduction) reactions, the anaerobic reaction mixture contained 0.25 mM NAD(P)H, 18 mM acetaldehyde, 1 to 10 μl cell-free extract, and 800 μl of Assay Buffer. Decrease in absorbance at 340 nm caused by NAD(P)H oxidation was monitored by an Agilent 8453 UV–vis spectrophotometer with Peltier controlled heating set at 55°C inside an anaerobic chamber. Protein concentration was determined by using the Bradford method [33].

### Amino acid analysis

Amino acid analysis was carried out by AminoAcids.com (St. Paul, MN, USA). Samples were prepared for analysis by appropriate dilution with a deproteinizing solution (13.5% w/v 5-sulfosalicylic acid hydrate), L-2-amino-3-guanidinopropionic acid hydrochloride, and eluent lithium buffer. The mixture was vortexed, micro-centrifuged, and filtered through a 0.2-μm filter. Amino acid analysis was performed on a Hitachi Model L-8900 HPLC with 10-cm cation exchange columns, four sequential lithium-based eluents (Hitachi and Pickering Laboratories), and lithium hydroxide for column regeneration. Absorbance was measured at 440 and 570 nm following post-column color development by ninhydrin reagent at 135°C. Data are an average of three independent biological replicates. AminoAcids.com reported that an unknown peak overlapped with the asparagine peak, preventing quantification in both the *ΔhydG* and *ΔhydGΔech* samples. As the ninhydrin method detects primary and secondary amines, the interfering peak presumably represents an as yet unidentified amino compound.

### Identification of mutation in alcohol dehydrogenase gene in *∆hydG* strain

The *adhE* region was PCR amplified as 982-bp product using primers XD520 and XD521 (Additional file 3). The primer region was 70 bp away from the D494G mutation [6], which was sequenced and confirmed with primer XD560 (Additional file 4).

## Additional files

Additional file 1:
**Confirmation of** ***C. thermocellum*** **deletion mutants.** A) Three primer sets were used for confirmation of the *hydG* deletion. Primer set (P1) and (P2) amplified 863-bp and 729-bp fragments of the *hydG*, respectively, which are present in the wild type but absent in *C. thermocellum ∆hydG*. Primer set (P3) amplifies the 3,500-bp region of the wild-type locus, while amplification from *∆hydG* mutant results in a 2,100-bp fragment. B) Similar primer sets (P4, P5, and P6) are used to confirm deletion of *ech* in the *C. thermocellum ∆hydG* background. P4 and P5 amplify 701-bp and 721-bp regions of *ech*, respectively, which are present in the *∆hydG* and wild-type strains but absent in the *∆hydG∆ech* strain. Primer set (P6) would amplify a 12,000-bp region of the wild-type locus and a 2,000-bp fragment in the *∆hydG∆ech* strain. C) PCR confirmation of deletion of *hydG* and *ech*. Lane m, DNA ladder with molecular weights noted (in kilobases); lane a, *∆hydG∆ech* template; lane b, *∆hydG* template; lane c, *C. thermocellum* wild-type template; lane d, no template PCR control. Additional file 2:
**Maximum optical density (OD) attained by wild-type and mutant strains of** ***C. thermocellum.*** (Left) rich and (right) minimal medium supplemented with 0 to 5% (v/v) added ethanol. Symbols: Open black squares, *C. thermocellum* wild type; closed gray squares, ethanol-tolerant control *C. thermocellum adhE**(EA); closed blue triangles, *C. thermocellum ∆hydG;* and open red triangles, *C. thermocellum ∆hydG∆ech*. Error bars represent one standard deviation. Additional file 3:
**Annotated plasmid sequences of pAMG275 and pAMG278.**
Additional file 4:
**List of primers used in this study.**

## Abbreviations

ADH: alcohol dehydrogenase
8AZH: 8-azahypoxanthine
CBP: consolidated bioprocessing
FUdR: 5-fluoro-2′-deoxyuridine
MOPS: 3-morpholino-propane-1-sulfonic acid
TM: thiamphenicol
