# Supplementary figures and images for: Elimination of hydrogenase active site assembly blocks H2 production and increases ethanol yield in Clostridium thermocellum
Source: Biotechnol Biofuels. 2015 Feb 12;8:20. doi: 10.1186/s13068-015-0204-4 (PMC4355364; doi:10.1186/s13068-015-0204-4)

Additional File 1.

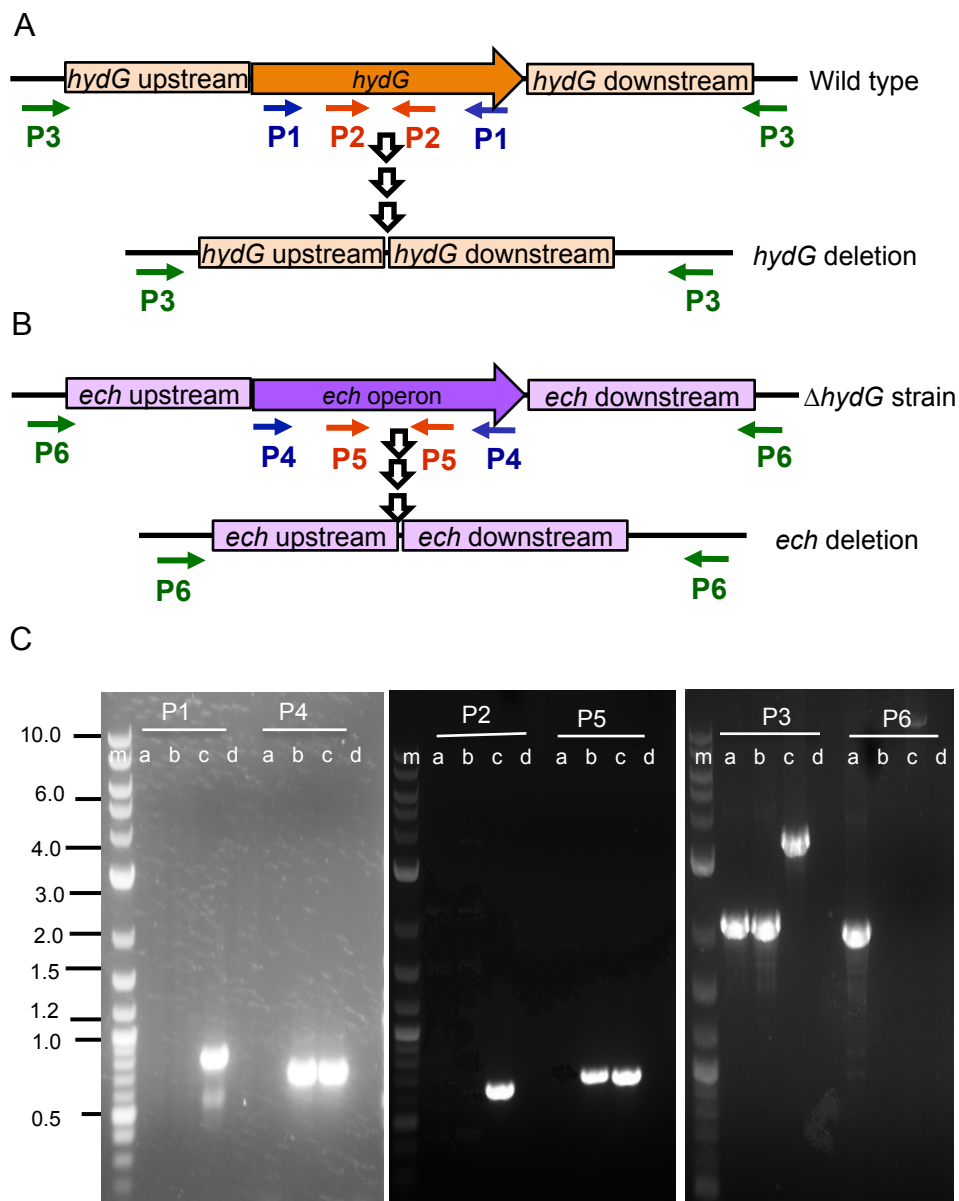

Additional File 2.

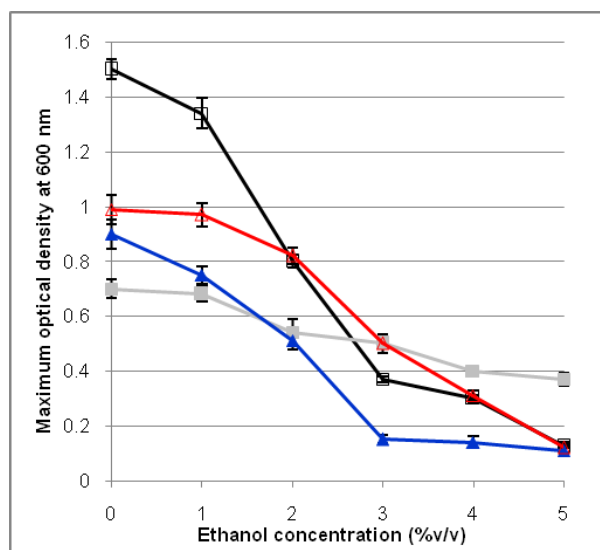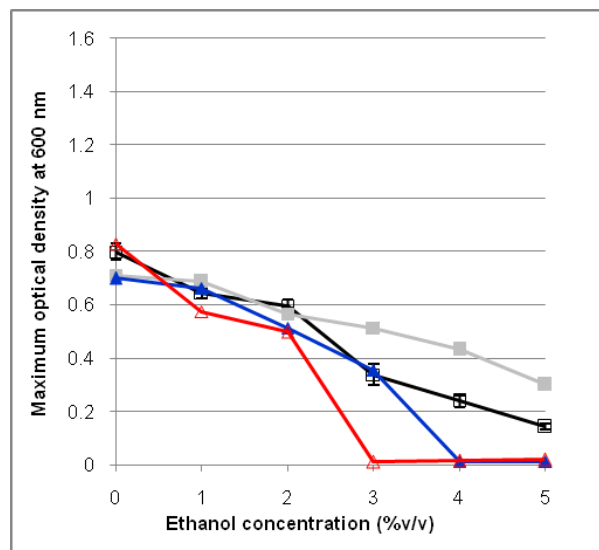

Supplement: Additional file 1: — Confirmation of C. thermocellum deletion mutants. A) Three primer sets were used for confirmation of the hydG deletion. Primer set (P1) and (P2) amplified 863-bp and 729-bp fragments of the hydG, respectively, which are present in the wild type but absent in C. thermocellum ∆hydG. Primer set (P3) amplifies the 3,500-bp region of the wild-type locus, while amplification from ∆hydG mutant results in a 2,100-bp fragment. B) Similar primer sets (P4, P5, and P6) are used to confirm deletion of ech in the C. thermocellum ∆hydG background. P4 and P5 amplify 701-bp and 721-bp regions of ech, respectively, which are present in the ∆hydG and wild-type strains but absent in the ∆hydG∆ech strain. Primer set (P6) would amplify a 12,000-bp region of the wild-type locus and a 2,000-bp fragment in the ∆hydG∆ech strain. C) PCR confirmation of deletion of hydG and ech. Lane m, DNA ladder with molecular weights noted (in kilobases); lane a, ∆hydG∆ech template; lane b, ∆hydG template; lane c, C. thermocellum wild-type template; lane d, no template PCR control. [file 13068_2015_204_MOESM1_ESM.pdf]

Additional File 2.

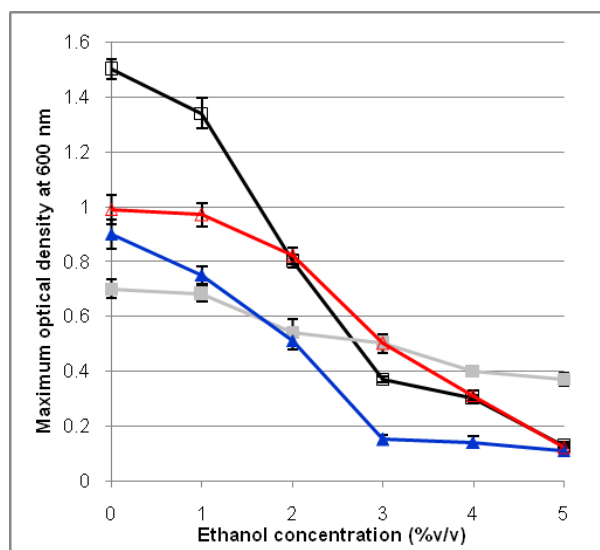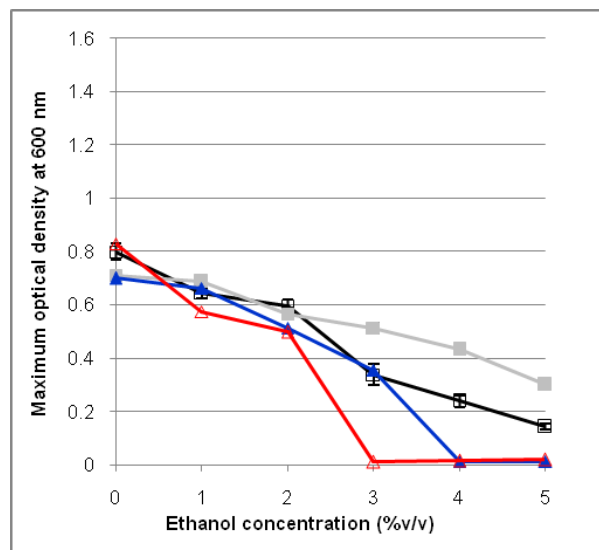

Supplement: Additional file 2: — Maximum optical density (OD) attained by wild-type and mutant strains of C. thermocellum. (Left) rich and (right) minimal medium supplemented with 0 to 5% (v/v) added ethanol. Symbols: Open black squares, C. thermocellum wild type; closed gray squares, ethanol-tolerant control C. thermocellum adhE*(EA); closed blue triangles, C. thermocellum ∆hydG; and open red triangles, C. thermocellum ∆hydG∆ech. Error bars represent one standard deviation. [file 13068_2015_204_MOESM2_ESM.pdf]
