# Supplementary material for: Elimination of hydrogenase active site assembly blocks H2 production and increases ethanol yield in Clostridium thermocellum
Source: Biotechnol Biofuels. 2015 Feb 12;8:20. doi: 10.1186/s13068-015-0204-4 (PMC4355364; doi:10.1186/s13068-015-0204-4)
Supplement: Additional file 4: — List of primers used in this study. [file 13068_2015_204_MOESM4_ESM.pdf]

Additional File 4. List of primers used in this study.

Primer name	Sequence, 5'→3'
P1- forward	GGGGATATTCAAATGGTTGA
P1/P2- reverse	GAACAGATATGGTGTGCGGC
P2-forward	CCTGGGAGAAGTTGCAAAGC
P3-forward	GCTGTAAGTCTTCGGTGAGAGTT
P3- reverse	CTCACTTTTGTAGAATCCACACCT
P4- forward	AATTTTTAGCAAATGCGGGG
P4- reverse	CCCTTTCAATGAAGCTTGCC
P5-forward	TTCTGGCTTCTCAACATTAATGG
P5- reverse	CGGTAAGAATCAGAGAAGTTTTTCA
P6-forward	AGCGCGGATATATGATGGC
P6- reverse	CGTAAGCGAAATTGAAAAGGAA
XD520	TTCTTCGTTATTCTTGCCCT
XD521	TTGTCTGCCTGTTGCATT
XD560	TGCCTTCTGTCCCATCTT
